# The Passing of Simmons

**Published:** 1911-09

**Authors:** 


THE PASSING OF SIMMONS.—So the boss is down and out as Secretary of the American Medical Association; but he still runs the great *Octopus* and will develop a great publishing business. I hope we have heard the last of him, and that peace will dwell perpetually in the ranks. A Dr. Craig becomes Secretary.

